# Feelings and Beliefs

**Published:** 1891-12-05

**Authors:** 


### The Hospital

A Weekly Journal of

Science, Medicine, Nursing, and Philanthropy.

*For Hospitals, Asylums, Infirmaries, Dispensaries, Medical Practitioners, Students, Nurses, and the Charitable Public.*

Vol. XI.—No. 271.] [Dec. 5, 1891.

### Feelings and Beliefs.

In his rectorial address, at Glasgow University last week, Mr. A. J. Balfour affirmed the proposition that: "Society is founded, not upon criticism, but upon feelings and beliefs, and upon the customs and codes by which feelings and beliefs are, as it were, fixed and rendered stable. A community founded upon argument would soon be a community no longer. It would dissolve into its constituent elements."

This is a word of light. Coming from Mr. Balfour, who is himself pre-eminently a debater and a logician, it is worthy of earnest consideration as a philosophical theory, and, still more, as a basis of practice. There are two nations which stand over against each other both in geographical position, and in intellectual characteristics. The one is England; the other France. The Frenchman is a theoriser—a logician. He posits his theory first, and then tries to live up to it. That is what he has been constantly doing, at least, during the present and last centuries. The Englishman is not a man of abstractions. He does not always make a point of having a cut and dry theory for every great movement or small detail of life. It is his line rather to ask what is the practical duty to be done? What is the scheme to be entered upon for the opening out of a continent, the fighting of a long campaign, or the earning of a dinner after the breakfast has been eaten? Let him but see that, and he shoulders his rifle or his mattock, as the case may be, and sets about the work forthwith.

It is fortunate that we have practical objects of comparison before us, whereby to estimate the value of Mr. Balfour's position. England has undoubtedly lived by feelings and beliefs. France by theories and arguments. The practical results have been open for the inspection of all men during the last two centuries. England has been stable; France revolutionary. England has spread herself over every part of the earth; France has remained at home. England has trebled her population since the beginning of the century; France has increased at the slowest of snails' paces. Such are the practical results of these two contrasted ideals of life.

Ours is not a political journal. Nevertheless, we have to deal with a large kingdom, endowed with practical necessities, and with much power for good or harm. If any of the institutions of this country have owed their origin, vitality, and usefulness to "feelings and beliefs" rather than to mere argumentation, it is our voluntary and endowed hospitals. But there seems to be a disposition on the part of a section of the Press and the public to dig away the foundations of belief and feeling on which the hospitals are built, and to substitute as a basis the airy intangibilities of acrid criticism. The critics of this class have a very peculiar method of using their eyes. They are like keen Yorkshire corn merchants who, finding a single spot of smut in a granary of wheat, immediately pronounce the whole sample bad. The hospitals of London and the country treat with the utmost possible kindness, consideration, and skill, thousands of in-patients and out-patients every week. Concerning this work, colossal in magnitude and noble in character and result, not one word of praise is ever uttered by a certain section of the Press. But let a single person have to wait for half-an-hour before being treated, or let an inexperienced probationer nurse complain that she has had to do without jam for her breakfast, and down come the critics, like painted cannibals with poison-tipped spears, to attack and destroy every institution which goes by the name of hospital.

Justice—what is justice to critics like these? Responsibility—what is responsibility compared with a sensational paragraph in the fifth edition of an evening newspaper? If such paragraphs shut up the pockets of hundreds of unreflecting subscribers to hospitals, and so deprive thousands of bread-winners, delicate mothers, and sickly children of indispensable medical relief—what is that to the paragraph-monger? He has made his little sensation; and being generally a raw and inexperienced youth, he finds a gleeful satisfaction therein. He comes to no harm by the process: his dinner awaits him at the usual hour, and his cigar and whisky and water at bedtime do not fail.

It is said by easy-going persons that such criticism does no harm; that the "great heart of the public" is in its right place, and beats sound and true to all that is noble and good. This is all right, so far as it goes. It is not the public heart we are afraid of, but the public head. The British lion, though not distinguished for logic, has a head and a very good one. But that head has at least two peculiarities; it works slowly when it does work, and it hates working at all when work can be avoided. This is the opportunity of the youthful newspaper critic. In he rushes, armed with his few scraps of facts, and covered with that toughest of all mail, unconquerable self-assurance, and boldly proclaims—I am the man; lock up your cheque-books, and listen to me. The unreflecting, as we have said, do listen to him, and carefully lock away their cheque-books during the process. Of course, it is but for a time. They find him out more or less speedily, and then dismiss him with a metaphorical kick. But, whilst the grass is growing, the steed is starving; and whilst the kind-hearted public have been listening to the reckless and inconsequent criticisms of the paragraph-monger, they have forgotten to supply the daily resources for the daily necessities of twenty thousand poor and hungry sick.

For this is the great fact to be borne in mind; that to cut off the supplies of a large army, even for a day or two, is to produce confusion, disaster, and death. That is the case even with an army of men. But what, then, must it be with a vast and helpless contingent of sick persons, some of whom are men, but still more are women and children? It is ruin to such as these to have their needful supplies limited, even for a single day. When they apply at the doors of the hospital, they must be taken in. Whatever "argument" may say to the contrary, "feeling" says, admit them; and not only feeling, but experience and belief. They must be admitted. It would be intolerable to shut the door in their faces. But if they are admitted they must have beds: and must they not also have food, and medicine, and nurses, and doctors: and if they are to have all these things, as plain sense and right feeling say they must have, how are they to get them without money? And how is the money to be provided day by day if every little paragraph in a newspaper is to be accepted as an adverse verdict against hospitals, and to shut up the pockets of the subscribers?

No great work can be done with effect without steadiness and continuity. When a great lake high up in the mountains supplies a steady stream of water to the brook, the mill on the river's bank can grind its corn every day in the week and every week in the year. But where there is no lake in the mountains, and the river is supplied by a few little streamlets that flow only in showery weather, its bed is soon empty and dry, and the sounds of busy industry in the mill may be silent for weeks at a stretch. Is it not exactly thus with the work of the voluntary hospitals? They must have their lake in the mountains; their lake of steady and generous donors and annual subscribers. They cannot afford to depend upon occasional fitful showers. Moreover, it is economic insanity to ask them to do so. They have their large buildings, their paid servants, and their costly appliances; they have also a perpetual inflow of necessitous sick, whom it is socially economical and advantageous to cure. To let their healing mill stand idle on the river bank for want of a steady stream of monetary help is in the highest degree inconsequent, and as un-English as it is ridiculous.

Some people say they are waiting for the report of the Royal Commission before subscribing any more. That is like refusing to give an old and faithful horse a feed of corn until the veterinary surgeon pronounces on the morrow whether he can do a little gentle work for another year or two or is to receive his *coup de grace* at once. Poor old fellow: he has a faithful record behind him: no critical argument is required to teach us what to do: feeling tells us to give him his corn at once, and every other comfort we can think of until the word of fate comes from the surgeon's mouth. It is exactly so with our hospitals; they have served both the rich and the poor nobly, gloriously. Where is the man, without fault, who is fit to cast a stone at them? It is not our logic and our argumentation that have built them up, but our manly feeling and our deep-rooted faith. Our hospitals, like our world-wide Empire, have been founded on feeling and faith; and by feeling and faith they will stand, we trust, so long as the world endures.

